# Prevalence of hepatitis B virus infection in Kenya: A study nested in the Kenya Population-based HIV Impact Assessment 2018

**DOI:** 10.1371/journal.pone.0310923

**Published:** 2024-11-14

**Authors:** Raphael O. Ondondo, Jacques Muthusi, Violet Oramisi, Daniel Kimani, Missiani Ochwoto, Peter Young, Catherine Ngugi, Anthony Waruru, Jane Mwangi, Ann Chao, Megan Bronson, Trudy Dobbs, Lucy Ng’ang’a, Nancy Bowen, Appolonia Aoko, Paige A. Armstrong, Rashid Aman, Marc Bulterys

**Affiliations:** 1 Division of Global HIV&TB, Center for Global Health, US Centers for Disease Control and Prevention (CDC), Nairobi, Kenya; 2 National AIDS and STI Control Program (NASCOP), Ministry of Health, Nairobi, Kenya; 3 Kenya Medical Research Institute (KEMRI), Nairobi, Kenya; 4 Division of Global HIV&TB, Center for Global Health, US Centers for Disease Control and Prevention (CDC), Maputo, Mozambique; 5 Center for Global Health, U.S. National Cancer Institute, Bethesda, Maryland, USA—based in Nairobi, Kenya; 6 Division of Global HIV&TB, Center for Global Health, US Centers for Disease Control and Prevention (CDC), Atlanta, GA, United States of America; 7 National HIV Reference Laboratory, Ministry of Health, Nairobi, Kenya; 8 Division of Viral Hepatitis, US Centers for Disease Control and Prevention (CDC), Atlanta, GA, United States of America; 9 Chief Administrative Secretary, Ministry of Health, Nairobi, Kenya; University of Cincinnati College of Medicine, UNITED STATES OF AMERICA

## Abstract

**Background:**

Sub-Saharan Africa region bears the highest chronic hepatitis B virus (HBV) infection burden worldwide. National estimates of HBV burden are necessary for a viral hepatitis program planning. This study estimated the national prevalence of HBV infection in Kenya among people aged 15–64 years.

**Methods:**

Of 27,745 participants age 15–64 years in the Kenya Population-based HIV Impact Assessment (KENPHIA) 2018 household survey, we analyzed data for all persons living with HIV (PLHIV; n = 1,521) and a random sample of HIV-negative persons (n = 1,551), totaling to 3,072 participants. We tested whole blood samples for hepatitis B surface antigen (HBsAg) using Determine™ HBsAg rapid test and used population projections to estimate national disease burden. Pearson chi square was performed and the weighted prevalence proportions presented.

**Findings:**

Of the 3,072 participants,124 tested HBsAg positive, resulting in a weighted national HBV prevalence of 3.0% (95% CI: 2.2–3.9%). This translated to an HBV infection burden of 810,600 (95% CI: 582,700–1,038,600) persons age 15–64 years in Kenya. Distribution of HBV prevalence varied widely (p<0.001) by geography, ranging from 0.1% in Eastern Kenya regions to over 5% in northern and western Kenya. Prevalence of HBV infection was higher in PLHIV (4.7%; 95% CI: 3.3–6.0%) compared to HIV-negative persons (3.0%; 95% CI: 2.1–3.9%), and was highest among persons: age 45–54 years (6.4%; 95% CI: 3.3–9.5%), those who reported no formal education (10.7%; 95% CI: 5.1–16.4%), in polygamous marriages (6.8%; 95% CI: 1.7–11.8%), and in the lowest wealth quintile (5.3%; 95% CI: 2.8–7.7). When adjusted for covariates, lack of formal education (aOR = 4.2; 95% CI: 1.5–12.6) was significantly associated with HBV infection. In stratified analysis by HIV status, residing in rural areas and history of blood transfusion were independently associated with HBV infection among PLHIV, while lack of formal education and no history of blood transfusion were associated with HBV infection among HIV-negative participants (p<0.05).

**Interpretation:**

HBV prevalence among persons aged 15–64 years in Kenya was 3.0%. Higher prevalence was documented among persons without formal education, in the lowest wealth quintile, and those living in Kenya’s North-Eastern, Rift Valley-North and Nyanza regions. Targeted programmatic measures to strengthen interventions against HBV infections including newborn vaccination and treatment of infected adults to limit mother-to-child transmission, would be helpful in reducing burden of HBV-associated viral hepatitis.

## Introduction

Infection with hepatitis B virus (HBV) is a major global health problem, and over 30% of people worldwide have serological evidence of past or current infection [1]. Chronic HBV is one of the top 10 leading causes of death worldwide, accounting for an estimated 900,000 deaths annually due to HBV–associated liver diseases [2]. The World Health Organization (WHO) set a goal of eliminating viral hepatitis by 2030, with targets including: 90% reduction of new HBV infections, 65% reduction in HBV-related mortality, and 80% access to treatment for those eligible [2]. Despite a significant reduction in the global prevalence of HBV infection among children aged <5 years (from 4.9% during the pre-vaccination era to 0.9% in 2019), primarily due to infant HBV vaccination [3], the global burden of chronic HBV remains significant in adults [3]. Globally, approximately 296 million people were living with chronic HBV infection in 2019 [4], and as of 2015, 2.7 million were co-infected with HIV [2]. An additional 1.5 million people were newly infected with HBV in 2019, and 820,000 people died from HBV infection-related causes [4]. The Western Pacific and African regions remain the WHO regions estimated to have the highest HBV prevalence [2, 4]. In 2019, the African region had the highest burden of new HBV infections, accounting for over 65% of the 1.5 million new cases globally [4]. In 2015, an estimated 1.3 million deaths related to viral hepatitis occurred globally, of which 66% were attributed to complications from chronic HBV infection [2]. Fortunately, HBV infection is at least 90% vaccine-preventable [5].

Morbidity and mortality due to chronic HBV infection could be reduced through appropriate early screening for treatment eligibility and initiation of recommended antiviral therapy [6]. In 2019, about 10% of the estimated 296 million people with chronic HBV infection worldwide were aware of their infection status, of whom 22% were on treatment [4]. Therefore, to achieve the 2030 WHO goal of eliminating viral hepatitis [2], a combination of prevention strategies, including antiviral therapy for eligible patients, routine infant HBV immunization with one birth dose within 24 hours of birth followed by two to three additional doses in infancy [7], and catch-up HBV vaccination among adolescents and young adults in high-prevalence settings must be scaled up. However, access to available effective treatment and catch-up vaccination in most low-resource countries remains poor primarily due to limited availability of diagnostics, perceived high cost of medication [2], and complexity of current WHO treatment guidelines [8]. Lack of nationally representative data on HBV burden is a key limitation to clear understanding of where the need for HBV treatment is greatest, and thus hindering political commitment and scale-up of intervention programs. Consequently, many countries in sub-Saharan Africa (SSA), including Kenya, have made limited progress in implementing the WHO integrated strategy of triple elimination of mother-to-child transmission of HIV, HBV and syphilis [9]. This study estimated the national burden of current HBV infection in Kenya among adolescents and adults aged 15–64 years.

## Methods

**Fig 1:**
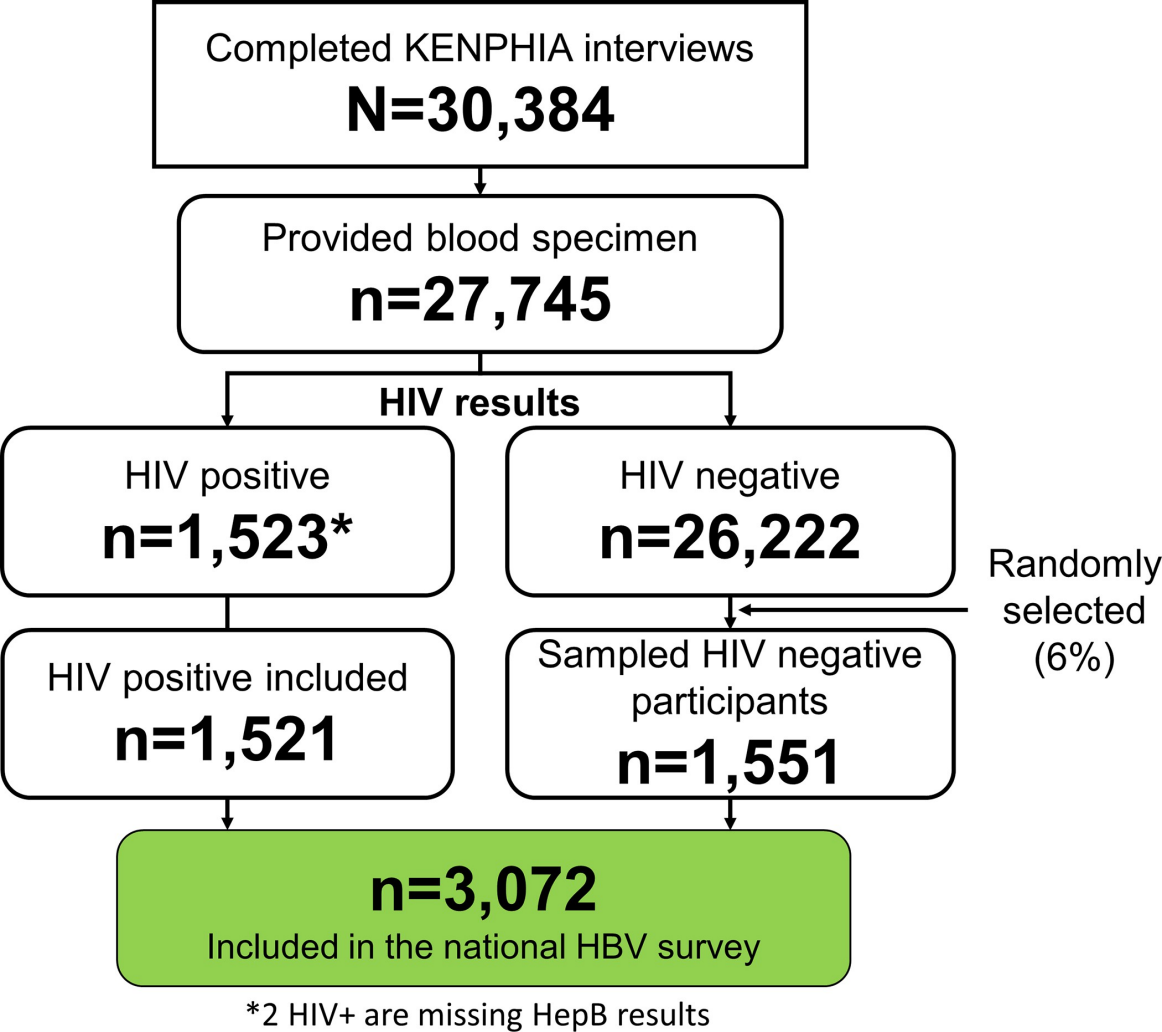
Sampling of participants for the national HBV study nested within the KENPHIA 2018 survey.

Population-based HIV Impact Assessments (PHIA) are being implemented across many countries supported by the U.S. President’s Emergency Plan for AIDS Relief (PEPFAR). During June 2018–February 2019, Kenya conducted a national survey to estimate the impact of investments in the HIV epidemic response called the Kenya Population-based HIV Impact Assessment (KENPHIA 2018). This sub-study estimating national HBV prevalence used the samples and data collected by KENPHIA survey. KENPHIA was a cross-sectional, household-based survey that used a two-stage, cluster-sampling design based on county HIV prevalence estimates. In 800 selected clusters, the survey targeted residents aged 0–64 years old, who slept in 16,918 sampled households the night before the survey [10]. A total of 27,745 participants aged 15–64 years old consented to survey participation and provided a blood specimen for biomarker testing. A structured social-behavioral questionnaire interview was administered [10]. All participants living with HIV (PLHIV) as identified in the survey, and a random sample of HIV-negative subjects at a rate designed to yield an overall self-weighting sample of 6% as detailed in the KENPHIA technical report [11], were selected for inclusion in the HBV prevalence sub-study (Fig 1). HBV testing in this study excluded children 0–14 years old because of expected low burden due to routine infant HBV immunization rolled out in Kenya in 2003/2004. Ethical approval was granted by the Kenya Medical Research Institute–Science and Ethics Research Unit (KEMRI-SERU-592), United States Centers for Disease Control and Prevention (CDC-IRB-7094) and Columbia University institutional review boards (IRB-AAAR7792 (Y05M01)).

All participants aged 18–64 years provided written informed consent. Parental/guardian permission was sought before obtaining written assent for adolescents aged 15–17 years. The structured questionnaire was administered by trained study staff using Open Data Kit (ODK) on a tablet. The questionnaire included modules on demographic characteristics, sexual and reproductive health, marital status, male circumcision, sexual activity, HIV/AIDS knowledge and attitudes, HIV testing and treatment history, history of TB and other diseases, and alcohol use among others.

Venous blood samples were collected for evaluation of HIV and current HBV infections. HIV testing was performed as per the Kenya National HIV Testing Services Guidelines [12], using Determine™ HIV-1/2 (Abbott Rapid Diagnostics, Lake Forest, Illinois, United States) and First Response™ HIV 1–2.0 (Premier Medical Corporation, Mumbai, India) HIV rapid test kits (S1 Fig), and results were provided onsite. At a satellite laboratory, HIV rapid positive results were confirmed using Geenius HIV 1/2 Confirmatory Assay (Bio-Rad Laboratories, Marnes-la-Coquette, France). The presence of hepatitis B surface antigen (HBsAg) was established per manufacturer’s instructions using Abbott’s Determine™ HBsAg rapid test kit that has a sensitivity and specificity of 98% and 100% respectively on whole blood samples [13]. Based on WHO’s recommendations for high HBV infection prevalence regions [14], no further confirmatory serological testing for HBV infection was performed in this study.

HBV and HIV rapid testing were carried out in the household, and results were given directly to participants. Those who tested positive were appropriately referred for further treatment eligibility assessments and care according to national guidelines. Polygamous marital status was defined as a relationship in which the man was married to or cohabiting with more than one woman. Wealth quintiles (lowest, second, third, fourth, and highest) were determined based on the wealth index as described elsewhere [15]. Due to small numbers in the highest wealth quintile, this was combined with the fourth quintile. Therefore, in this analysis, we presented wealth quintiles in four categories: lowest, second, third, and highest.

### Data analysis

Survey weights were calculated for probabilities of selection and adjusted for the interview, blood draw, HIV testing non-response, and under-coverage. After post-stratification, the weights were normalized to the survey sample populations. A detailed description of the survey weighting process is discussed in the Sampling and Weighting Technical Report [11]. The estimated HBV burden in the general population was based on projections for the 2018 *de facto* population by sex and age group [11]. Among PLHIV, HBV burden was based on the 1.3 million adults (15–64 years old) living with HIV in Kenya as estimated in 2018, and 96.0% (95% CI: 94.7%–97.3%) were on antiretroviral therapy (ART) against HIV [10]. Pearson chi-square test was used to test for differences by demographic characteristics and risk exposure categories of enrolled participants by sex. HBV prevalence was measured and presented as weighted proportions of participants who tested positive to hepatitis B surface antigen during the survey period.

To describe the geographical distribution of the HBV burden in Kenya, we used the ten regions of the National AIDS and STI Control Programme (NASCOP) described in the Kenya AIDS Indicator Survey (KAIS) of 2012 [16]. These NASCOP regions were categorized by HIV burden based on administrative regions (former provinces). Logistic regression modeling on weighted data was used to evaluate factors associated with current HBV infection, and multiple logistic regression modeling was performed to obtain estimates of adjusted odds ratios (aOR) and 95% confidence intervals (CI). Stratified logistic regression by HIV status was performed to identify independent factors for HBV infection in each group (PLHIV and HIV-negative separately). Analyses were conducted using Statistical Analysis Software (SAS) version 9.4 (SAS® 9.4 Base SAS. Cary, NC: SAS Institute Inc., 2014). Variables with p-values less than 0.05 or non-overlapping 95% confidence interval (CI) were considered statistically significant. Survey design parameters including survey weights, clustering, stratification, and variance replication methods were used in all analyses.

## Results

In this sub-study, a total of 3,072 (1,521 PLHIV and 1,551 HIV-negative) participants drawn from the KENPHIA 2018 survey were evaluated for HBV infection. The median age was 28.8 years, interquartile range (IQR): (20.7–39.3) years, and two-thirds of study participants were female (Table 1). The survey found that 64.0% (95% CI: 61.1–67.0%) of the study population were residents of rural settings, 50.9% reported being married or cohabiting, and only 33.4% (95% CI: 30.9–35.9%) reported having completed primary school education level (Table 1). Distribution of other sociodemographic characteristics among males and females did not differ significantly (based on overlapping CIs) (Table 1).

**Table 1:** Characteristics of KENPHIA 2018 survey participants sampled for Kenya HBV sub-study.

	Male (n = 1084)			Female (n = 1988)			Total (N = 3072)		
Characteristic	Total (n)	Weighted (%)	95% CI	Total (n)	Weighted (%)	95% CI	Total (N)	Weighted (%)	95% CI
**Residence**									
Urban	361	36.3	(32.1–40.6)	726	35.6	(31.7–39.6)	1087	36.0	(33.0–38.9)
Rural	723	63.7	(59.4–67.9)	1262	64.4	(60.4–68.3)	1985	64.0	(61.1–67.0)
**Ten-year age groups**									
15–24	260	36.3	(36.3–36.3)	402	34.9	(34.9–34.9)	662	35.6	(35.6–35.6)
25–34	233	27.1	(27.1–27.1)	614	28.7	(28.7–28.7)	847	27.9	(27.9–27.9)
35–44	253	18.5	(18.5–18.5)	468	18.8	(18.8–18.8)	721	18.7	(18.7–18.7)
45–54	207	11.5	(11.5–11.5)	320	11.1	(11.1–11.1)	527	11.3	(11.3–11.3)
55–64	131	6.6	(6.6–6.6)	184	6.5	(6.5–6.5)	315	6.6	(6.6–6.6)
**Marital status (n = 2983)**									
Never married/ cohabited	313	42.0	(39.4–44.6)	403	29.8	(27.4–32.2)	716	35.8	(34.1–37.6)
Married/cohabiting—monogamous	591	46.8	(43.6–50.0)	798	46.5	(43.5–49.6)	1389	46.6	(44.4–48.9)
Married/cohabiting—polygamous	49	2.1	(1.2–3.1)	175	6.5	(5.1–7.8)	224	4.3	(3.5–5.2)
Divorced or separated or Widowed	120	7.8	(5.7–9.8)	534	14.3	(12.3–16.2)	654	11.1	(9.6–12.5)
**Education level (n = 3071)**									
No Formal Education	60	4.9	(3.1–6.6)	180	8.3	(6.6–10.0)	240	6.6	(5.4–7.9)
Incomplete Primary	484	37.0	(33.2–40.8)	1106	42.5	(39.4–45.6)	1590	39.8	(37.2–42.4)
Completed Primary	360	35.4	(31.6–39.3)	504	31.5	(28.6–34.3)	864	33.4	(30.9–35.9)
**Wealth quintile (n = 3071)**									
Highest	318	34.6	(30.2–39.0)	601	36.9	(33.2–40.6)	919	35.8	(32.8–38.8)
Third	252	21.5	(18.1–24.9)	464	21.4	(18.4–24.4)	716	21.4	(19.0–23.8)
Second	270	23.2	(19.8–26.5)	466	20.4	(17.8–23.0)	736	21.8	(19.5–24.1)
Lowest	244	20.8	(16.8–24.7)	456	21.3	(18.5–24.1)	700	21.0	(18.4–23.6)
**Religion (n = 3071)**									
Roman Catholic	241	19.7	(16.4–23.0)	386	18.1	(15.4–20.7)	627	18.9	(16.8–20.9)
Protestant/Other Christians	712	67.1	(63.0–71.2)	1451	71.5	(68.4–74.7)	2163	69.3	(66.7–72.0)
Muslim	67	7.8	(4.9–10.8)	107	8.1	(6.3–9.9)	174	8.0	(6.1–9.9)
No Religion	45	3.6	(2.2–5.0)	23	1.5	(0.5–2.6)	68	2.6	(1.7–3.4)
Other	18	1.6	(0.5–2.7)	21	0.7	(0.1–1.3)	39	1.2	(0.5–1.8)
**Ever had blood transfusion (n = 3068)**									
Yes	40	2.9	(1.6–4.2)	154	5	(3.7–6.3)	194	4	(3.1–4.9)
No	1041	97.1	(95.8–98.4)	1833	95	(93.7–96.3)	2874	96	(95.1–96.9)
**Pregnancy Status**									
Pregnant				78	4.9	(3.4–6.3)			
Non-pregnant				1887	93.4	(91.7–95.1)			
**HIV Status**									
Positive	423	3.1	(2.7–3.5)	1098	6.6	(6.0–7.1)	1521	4.9	(4.5–5.3)
Negative	661	96.9	(96.5–97.3)	890	93.4	(92.9–94.0)	1551	95.1	(94.7–95.5)

Note: n = 3072 unless stated. For n<3072, the difference accounts for missing data due to non-response to the specific survey question

### Prevalence of hepatitis B infection

Of the 3072 participants, 124 tested positive for HBsAg resulting in a weighted national HBV prevalence of 3.0% (95% CI: 2.2–3.9%) in Kenya among persons aged 15–64 years old (Table 2). HBV prevalence was higher among PLHIV (4.7%; 95% CI: 3.3–6.0%) compared to HIV-negative participants (3.0%; 95% CI: 2.1–3.9%). These translated to an estimated 810,600; (95% CI: 582,700–1,038,600) persons living with HBV in Kenya among 15–64 years old in the general population and 61,000 (95% CI: 42,400–79,600) among PLHIV. The proportion of HBV-coinfected PLHIV on ART was 72.7% (95% CI: 61.4–82.3%). The prevalence of HBV infection was similar in men (3.3%; 95% CI: 2.1–4.6%) and women (2.8%; 95% CI: 1.7–3.8%), while HIV-positive participants residing in rural Kenya had a higher prevalence of HBV infection (5.7%; 95% CI: 3.8–7.7%) compared to 2.8% (95% CI: 1.6–4.1%) among their counterparts residing in urban Kenya (Table 2). The prevalence was lowest at 1.9% (95% CI: 0.6–3.2%) among participants aged 15–24 years and highest at 6.4% (95% CI: 3.3–9.5%) among those aged 45–54 years (Table 2). However, none of these variations in the prevalence of HBV infection by sex or age was statistically significantly different.

**Table 2:** Prevalence of hepatitis B surface antigen (HBsAg) positivity among KENPHIA 2018 survey participants sampled for Kenya HBV sub-study, by sex and HIV status (N = 3072).

Characteristic	By Sex				By HIV Status				Total	
	Male (n = 1084)		Female (n = 1988)		Positive (n = 1521)		Negative (n = 1551)		(N = 3072)	
	Unweighted n/N	HBV positive Weighted % (95% CI)	Unweighted n/N	HBV positive Weighted % (95% CI)	Unweighted n/N	HBV positive Weighted % (95% CI)	Unweighted n/N	HBV positive Weighted % (95% CI)	Unweighted n/N	HBV positive Weighted % (95% CI)
**Overall**	49/1084	3.3 (2.1–4.6)	75/1988	2.8 (1.7–3.8)	77/1521	4.7 (3.3–6.0)	47/1551	3.0 (2.1–3.9)	124/3072	3.0 (2.2–3.9)
**Sex**										
Male					26/423	5.1 (2.6–7.6)	23/661	3.3 (2.0–4.6)	49/1084	3.3 (2.1–4.6)
Female					51/1098	4.5 (3.0–6.0)	24/890	2.6 (1.5–3.8)	75/1988	2.8 (1.7–3.8)
**HIV Status**										
Positive	26/423	5.1 (2.6–7.6)	51/1098	4.5 (3.0–6.0)					77/1521	4.7 (3.3–6.0)
Negative	23/661	3.3 (2.0–4.6)	24/890	2.6 (1.5–3.8)					47/1551	3.0 (2.1–3.9)
**Residence**										
Urban	15/361	2.2 (0.4–3.9)	23/726	2.6 (0.6–4.7)	26/604	2.8 (1.6–4.1)	12/483	2.4 (0.8–3.9)	38/1087	2.4 (0.9–3.9)
Rural	34/723	4.0 (2.2–5.7)	52/1262	2.8 (1.7–3.9)	51/917	5.7 (3.8–7.7)	35/1068	3.3 (2.2–4.4)	86/1985	3.4 (2.3–4.4)
**Ten-year age groups**										
15–24	7/260	3.1 (0.7–5.5)	7/402	0.7 (0.0–1.5)	5/139	2.5 (0.0–5.2)	9/523	1.9 (0.6–3.2)	14/662	1.9 (0.6–3.2)
25–34	9/233	1.8 (0.0–4.0)	29/614	4.2 (1.5–7.0)	26/450	5.3 (2.7–7.8)	12/397	3.0 (1.0–5.0)	38/847	3.1 (1.2–5.0)
35–44	13/253	3.2 (0.2–6.3)	16/468	3.1 (0.7–5.4)	20/430	4.3 (2.1–6.6)	9/291	3.0 (0.9–5.1)	29/721	3.1 (1.2–5.1)
45–54	12/207	7.6 (1.9–13.3)	17/320	5.1 (1.9–8.4)	16/331	3.9 (1.8–6.0)	13/196	6.6 (3.2–10.0)	29/527	6.4 (3.3–9.5)
55–64	8/131	3.5 (0.0–8.1)	6/184	2.7 (0.0–5.8)	10/171	7.9 (1.7–14.0)	4/144	2.7 (0.0–5.7)	14/315	3.1 (0.3–5.8)
**Marital status (n = 2983)**										
Never married/ cohabited	7/313	2.6 (0.7–4.5)	9/403	1.8 (0.0–3.8)	5/199	1.4 (0.0–2.7)	11/517	2.3 (0.9–3.7)	16/716	2.3 (0.9–3.6)
Married/cohabiting—monogamous	30/591	3.5 (1.6–5.4)	29/798	2.8 (1.2–4.3)	35/646	5.4 (3.3–7.5)	24/743	3.0 (1.6–4.4)	59/1389	3.1 (1.8–4.4)
Married/cohabiting—polygamous	4/49	5.6 (0.0–15.7)	12/175	7.2 (1.4–12.9)	11/145	6.7 (1.7–11.7)	5/79	6.8 (1.2–12.3)	16/224	6.8 (1.7–11.8)
Divorced or separated or Widowed	7/120	5.0 (0.0–10.8)	21/534	2.2 (0.6–3.7)	23/479	4.3 (2.2–6.3)	5/175	3.0 (0.4–5.6)	28/654	3.2 (0.9–5.4)
**Education level (n = 3071)**										
No formal education	9/60	12.2 (0.8–23.5)	12/180	9.9 (3.7–16.1)	9/121	7.7 (1.2–14.3)	12/119	10.9 (4.9–16.9)	21/240	10.7 (5.1–16.4)
Incomplete Primary	20/484	3.9 (1.7–6.2)	45/1106	2.9 (1.3–4.5)	44/975	4.3 (2.7–6.0)	24/750	3.1 (1.7–4.5)	68/1725	3.2 (1.9–4.5)
Completed Primary	17/360	2.1 (0.4–3.8)	11/504	1.6 (0.0–3.3)	24/424	4.6 (2.5–6.6)	11/682	1.7 (0.6–2.8)	35/1106	1.8 (0.8–2.8)
**Wealth quintile (n = 3071)**										
Highest	10/318	1.2 (0.0–2.7)	17/601	2.0 (0.3–3.8)	20/414	4.4 (2.1–6.7)	7/505	1.5 (0.3–2.7)	27/919	1.6 (0.4–2.8)
Third	11/252	3.8 (0.6–6.9)	12/464	2.3 (0.2–4.3)	13/370	3.9 (1.6–6.2)	10/346	3.0 (1.0–4.9)	23/716	3.0 (1.2–4.9)
Second	11/270	4.6 (1.2–8.0)	18/466	1.8 (0.1–3.5)	18/377	4.8 (2.2–7.5)	11/359	3.2 (1.1–5.2)	29/736	3.3 (1.3–5.2)
Lowest	17/244	5.0 (1.3–8.8)	28/456	5.5 (2.5–8.5)	26/359	5.9 (2.0–9.8)	19/341	5.2 (2.6–7.8)	45/700	5.3 (2.8–7.7)
**Religion (n = 3071)**										
Roman Catholic	14/241	3.4 (0.3–6.6)	16/386	2.2 (0.4–4.0)	22/322	5.3 (1.5–9.1)	8/305	2.7 (0.4–4.9)	30/627	2.8 (0.7–5.0)
Protestant/Other Christian	28/712	2.7 (1.3–4.1)	47/1451	2.5 (1.2–3.7)	48/1092	4.5 (3.0–5.9)	27/1071	2.5 (1.5–3.5)	75/2163	2.6 (1.6–3.5)
Muslim	2/67	3.8 (0.0–9.8)	8/107	6.4 (1.4–11.5)	3/53	4.5 (0.0–12.2)	7/121	5.2 (1.1–9.3)	10/174	5.2 (1.2–9.2)
No Religion	3/45	11.1 (0.0–25.1)	2/23	5.0 (0.0–15.1)	1/31	2.7 (0.0–8.1)	4/37	9.5 (0.0–20.3)	5/68	9.2 (0.0–19.5)
Other	2/18	8.1 (0.0–26.1)	2/21	1.1 (0.0–2.6)	3/23	9.3 (0.0–20.5)	1/16	5.6 (0.0–18.7)	4/39	5.8 (0.0–18.0)
**Ever had blood transfusion (n = 3068)**										
Yes	1/40	0.2 (0.0–0.7)	11/154	2.7 (1.7–3.8)	11/129	9.0 (3.3–14.6)	1/65	1.1 (0.8–1.5)	12/194	1.8 (1.1–2.5)
No	48/1041	3.4 (2.1–4.7)	64/1833	2.8 (1.7–3.9)	66/1390	4.3 (2.9–5.7)	46/1484	3.0 (2.1–4.0)	112/2874	3.1 (2.2–4.0)
**Pregnant during study (n = 1965)**										
Yes			3/78	4.2 (0.0–10.0)	1/39	1.4 (0.0–3.6)	2/39	4.3 (0.0–10.5)	3/78	4.2 (0.0–10.0)
No			71/1887	2.7 (1.6–3.7)	50/1048	4.7 (3.1–6.2)	21/839	2.5 (1.4–3.7)	71/1887	2.7 (1.6–3.7)
**Geographic Region**										
Nairobi	1/65	0.1 (0.0–0.4)	1/94	1.5 (0.0–4.5)	1/53	1.7 (0.0–5.4)	1/106	0.7 (0.0–2.2)	2/159	0.8 (0.0–2.2)
Central	7/110	5.4 (0.4–10.4)	2/165	1.2 (0.0–3.5)	3/85	3.2 (0.0–7.1)	6/190	3.3 (0.4–6.1)	9/275	3.3 (0.5–6.0)
Nyanza	15/260	5.0 (1.5–8.5)	26/575	4.1 (0.1–8.2)	34/631	4.9 (3.1–6.6)	7/204	4.5 (1.4–7.7)	41/835	4.6 (1.8–7.3)
Rift Valley-North	8/138	2.9 (0.0–6.3)	16/242	6.7 (2.7–10.8)	13/172	10.6 (2.1–19.1)	11/208	4.7 (1.9–7.5)	24/380	5.0 (2.3–7.7)
Rift Valley-South	4/117	3.7 (0.2–7.1)	4/184	0.7 (0.0–2.1)	3/133	1.2 (0.0–2.5)	5/168	2.4 (0.2–4.6)	8/301	2.4 (0.3–4.5)
Eastern-North	0/14	. (.—.)	2/22	0.2 (0.0–0.5)	2/16	6.4 (0.0–15.4)	0/20	. (.—.)	2/36	0.1 (0.0–0.2)
Eastern-South	6/162	3.6 (0.8–6.4)	8/297	0.8 (0.0–1.8)	8/175	5.0 (1.4–8.7)	6/284	2.1 (0.5–3.6)	14/459	2.2 (0.7–3.7)
Western	5/126	2.2 (0.0–6.1)	6/225	1.9 (0.0–4.3)	8/150	4.7 (1.1–8.2)	3/201	1.9 (0.0–4.9)	11/351	2.0 (0.0–4.9)
North-Eastern	0/15	. (.—.)	2/16	15.8 (0.0–38.9)	0/2	. (.—.)	2/29	7.6 (0.0–17.8)	2/31	7.6 (0.0–17.8)
Coast	3/77	3.5 (0.0–9.2)	8/168	3.5 (1.3–5.8)	5/104	3.6 (0.0–8.6)	6/141	3.5 (0.5–6.6)	11/245	3.5 (0.6–6.4)

Note: n = 3072 unless stated. For n<3072, the difference accounts for missing data due to non-response to the specific survey question

The prevalence of HBV infection was 6-times higher among participants who reported no formal education (10.7%; 95% CI:5.1–16.4%) than those who reported completing primary education level (1.8%; 95% CI: 0.8–2.8%). Higher prevalence of HBV infection was found among participants who reported being in polygamous married/cohabiting relationships (6.8%; 95% CI: 1.7–11.8%) than those who reported single and never married (2.3%; 95% CI: 0.9–3.6%) and those in the lowest wealth quintile (5.3%; 95% CI: 2.8–7.7%) than those in the highest wealth quintile (1.6%; 95% CI: 0.4–2.8%), although with overlapping CIs (Table 2). The prevalence of HBV infection was higher among participants without history of blood transfusion at 3.1% (2.2–4.0%), compared to 1.8% (1.1–2.5%) among those who reported ever having a blood transfution prior to study participation. However, HIV-positive participants with a history of blood transfusion had a higher prevalence of HBV infection at 9.0% (3.3–14.6%), compared to 1.1% (0.8–1.5) found in their HIV-negative counterparts (Table 2).

**Fig 2:**
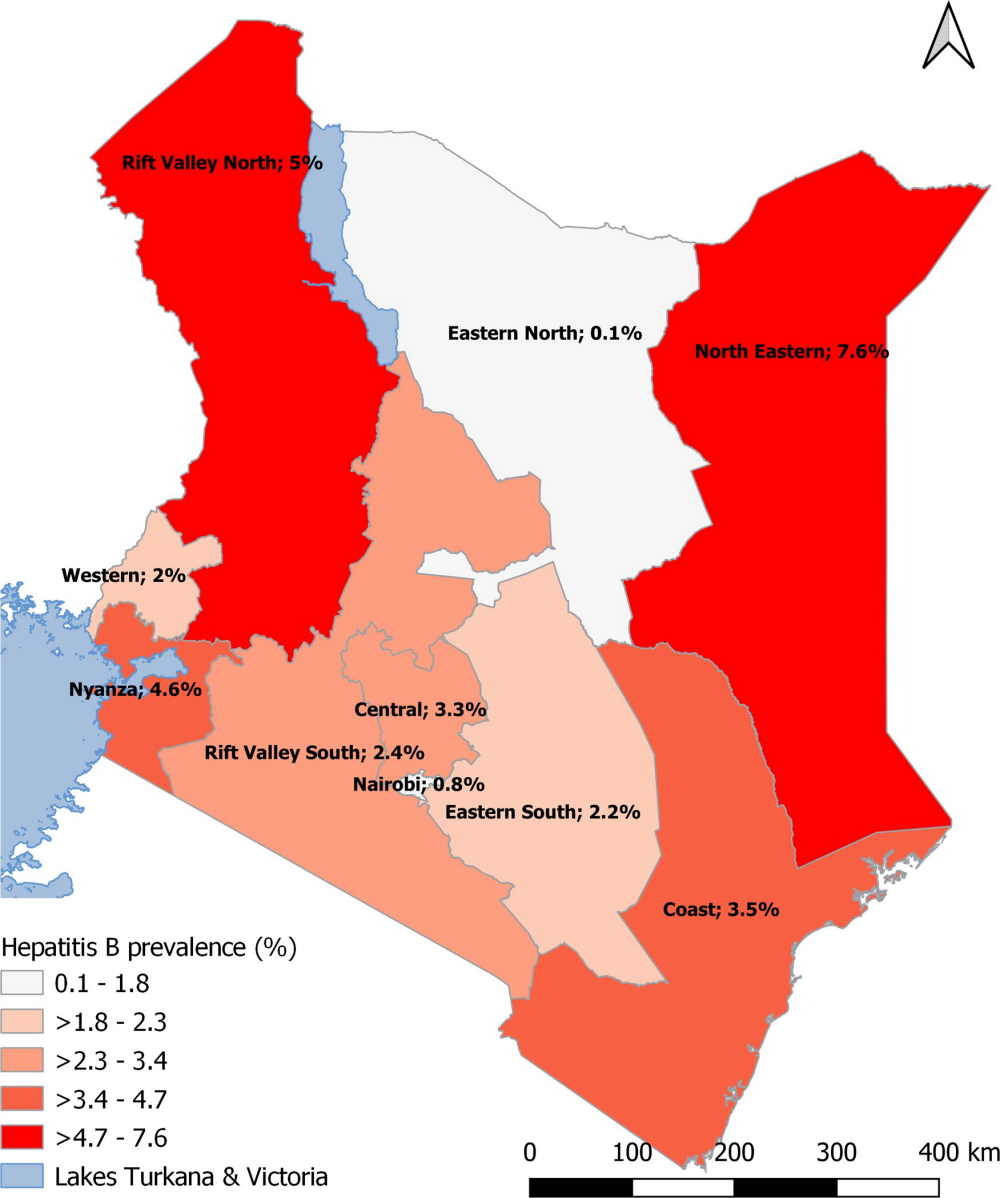
HBV prevalence by NASCOP regions, KENPHIA 2018 survey.

The prevalence of HBV infection in Kenya varied widely by geographic region (Fig 2), ranging from 0.1% (95% CI: 0.0–0.2%) in some Eastern regions of Kenya to 7.6% (95% CI: 0.0–17.8%) in North-Eastern regions (Table 2). The next top two HBV high-prevalence regions after North-Eastern were Rift Valley North (5.0%; 95% CI: 2.3–7.7%) and Nyanza (4.6%; 95% CI: 1.8–7.3%) in western Kenya (Table 2). Higher HBV prevalence among men was observed in Central (5.4%; 95% CI: 0.4–10.4%) and Nyanza (5.0%; 95% CI: 1.5–8.5%), and among women in Rift Valley-North (6.7%; 95% CI: 2.7–10.8%) and Nyanza (4.1%; 95% CI: 0.1–8.2%) regions, compared to other regions in Kenya (Table 2).

### Factors associated with hepatitis B infection

Table 3 presents both bivariate and multivariate analysis of factors associated with HBV infection. In multivariate logistic regression analysis, lack of formal education (aOR = 4.2; 95% CI: 1.5–12.6) remained the only independent factor found to be associated with HBV infection (Table 3). HIV infection (aOR = 1.5; 95% CI: 0.9–2.5), age 45–54 years (aOR = 2.6; 95% CI: 0.9–8.4), married/cohabiting polygamous marital status (aOR = 1.2; 95% CI: 0.4–4.3), lowest wealth quintile (aOR = 2.6; 95% CI: 1.0–6.6) and history of blood transfusion (aOR = 1.9; 95% CI: 1.0–3.7) were no longer associated with HBV infection as had been observed in bivariate analysis (Table 3). Additionally, none of the other socio-demographic characteristics was associated with HBV infection (Table 3).

**Table 3:** Factors associated with hepatitis B prevalence among participants in the KENPHIA 2018 survey HBV sub-study, N = 3072.

Characteristic	Total	HBV positive, n (Weighted %)	OR (95% CI)	P-value	Global P-Value	aOR (95% CI)	P-value	Global P-value
**HIV Status**								
Negative	1551	47 (3.0)	ref					
Positive	1521	77 (4.7)	1.6 (1.0–2.5)	0.024	0.024	1.5 (0.9–2.5)	0.076	0.076
**Residence**								
Urban	1087	38 (2.4)	ref					
Rural	1985	86 (3.4)	1.4 (0.7–2.8)	0.292	0.292	0.9 (0.4–1.8)	0.707	0.707
**Sex**								
Female	1988	75 (2.8)	ref					
Male	1084	49 (3.3)	1.2 (0.7–2.1)	0.453	0.453	1.4 (0.7–2.5)	0.294	0.294
**Ten-year age groups**								
15–24	662	14 (1.9)	ref					
25–34	847	38 (3.1)	1.7 (0.6–4.4)	0.276	0.042	1.6 (0.5–5.3)	0.413	0.286
35–44	721	29 (3.1)	1.7 (0.6–4.4)	0.26		1.7 (0.5–6.0)	0.349	
45–54	527	29 (6.4)	3.6 (1.5–8.7)	0.003		2.6 (0.9–8.4)	0.077	
55–64	315	14 (3.1)	1.7 (0.5–5.8)	0.41		1.2 (0.2–6.4)	0.811	
**Religion (n = 3071)**								
Protestant/Other Christians	2163	75 (2.6)	ref					
Muslim	174	10 (5.2)	2.1 (0.8–5.3)	0.113	0.255	1.2 (0.4–4.0)	0.733	0.808
No Religion	68	5 (9.2)	3.8 (0.9–16.8)	0.06		2.5 (0.6–11.4)	0.210	
Other	39	4 (5.8)	2.3 (0.0–280.9)	0.716		2.1 (0.0–295.8)	0.753	
Roman Catholic	627	30 (2.8)	1.1 (0.5–2.5)	0.805		1.1 (0.5–2.5)	0.817	
**Marital status (n = 2983)**								
Never married/ cohabited	716	16 (2.3)	ref					
Divorced or separated or widowed	654	28 (3.2)	1.4 (0.5–3.6)	0.469	0.134	0.7 (0.2–2.3)	0.502	0.607
Married/cohabiting—monogamous	1389	59 (3.1)	1.4 (0.7–3.0)	0.375		0.8 (0.3–2.0)	0.575	
Married/cohabiting—polygamous	224	16 (6.8)	3.1 (1.1–8.5)	0.019		1.2 (0.4–4.3)	0.734	
**Education level (n = 3071)**								
Complete Primary	1106	35 (1.8)	ref					
Incomplete Primary	1725	68 (3.2)	1.8 (0.8–3.8)	0.107	< .001	1.2 (0.5–2.7)	0.620	0.016
No formal education	240	21 (10.7)	6.6 (2.9–15.0)	< .001		4.2 (1.5–12.6)	0.006	
**Wealth quintile (n = 3071)**								
Highest	919	27 (1.6)	ref		0.035			0.134
Third	716	23 (3.0)	1.9 (0.8–4.6)	0.135		2.2 (0.9–5.0)	0.055	
Second	736	29 (3.3)	2.1 (0.8–5.3)	0.115		2.1 (0.8–5.4)	0.102	
Lowest	700	45 (5.3)	3.4 (1.4–8.0)	0.003		2.6 (1.0–6.6)	0.037	
**Ever had blood transfusion (n = 3068)**								
Yes	194	12 (1.8)	ref					
No	2874	112 (3.1)	1.7 (1.0–2.8)	0.025	0.025	1.9 (1.0–3.7)	0.054	0.054

Note

a) n = 3072 unless stated. For n<3072, the difference accounts for missing data due to non-response to the specific survey question

b) Table shows both bivariate (odds ratio—OR) and multivariate (adjusted odds ratio–aOR) analysis

In stratified analysis by HIV status (Table 4), residing in rural Kenya (aOR = 2.8; 95% CI: 1.2–6.5) and being married/cohabiting in polygamous relationships (aOR = 3.7; 95% CI: 1.0–13.7) were associated with increased likelihood of HBV infection among PLHIV. Additionally, PLHIV reporting no history of blood transfusion had decreased odds of HBV infection (aOR = 0.4; 95% CI: 0.2–0.9). On the contrary, neither residence nor marital status was associated with HBV infection among HIV-negative participants (Table 4). In this subpopulation, lowest wealth quintile (aOR = 2.5; 95% CI: 1.0–6.1), lack of formal education (aOR = 4.8; 95% CI: 1.5–15.7) and no blood transfusion history (aOR = 3.2; 95% CI: 1.6–6.6) were associated with significantly higher odds of HBV infection (Table 4).

**Table 4:** Factors associated with hepatitis B surface antigen (HBsAg) positivity among participants in the KENPHIA 2018 survey HBV sub-study, stratified by HIV status (N = 3072).

	HIV-positive participants—KENPHIA 2018, N = 1521								HIV-negative participants—KENPHIA 2018, N = 1551							
Characteristic	Total	HBV positive, n (Weighted %)	OR (95% CI)	P-value	Global P-value	aOR (95% CI)	P-value	Global P-value	Total	HBV positive, n (Weighted %)	OR (95% CI)	P-value	Global P-value	aOR (95% CI)	P-value	Global P-value
**Residence**																
Urban	604	26 (2.8)	ref						483	12 (2.4)	ref					
Rural	917	51 (5.7)	2.1 (1.2–3.7)	0.008	0.008	2.8 (1.2–6.5)	0.012	0.012	1068	35 (3.3)	1.4 (0.7–2.9)	0.359	0.359	0.8 (0.4–1.7)	0.544	0.544
**Sex**																
Female	1098	51 (4.5)	ref						890	24 (2.6)	ref					
Male	423	26 (5.1)	1.1 (0.6–2.0)	0.655	0.655	1.1 (0.6–2.3)	0.698	0.698	661	23 (3.3)	1.2 (0.7–2.2)	0.423	0.423	1.4 (0.7–2.7)	0.307	0.307
**Ten-year age groups**																
15–24	139	5 (2.5)	ref						523	9 (1.9)	ref					
25–34	450	26 (5.3)	2.1 (0.7–6.2)	0.147	0.353	2.8 (0.7–11.5)	0.127	0.344	397	12 (3.0)	1.6 (0.6–4.4)	0.337	0.038	1.6 (0.5–5.7)	0.431	0.234
35–44	430	20 (4.3)	1.7 (0.5–5.5)	0.325		1.9 (0.4–9.0)	0.376		291	9 (3.0)	1.6 (0.6–4.5)	0.312		1.7 (0.5–6.9)	0.312	
45–54	331	16 (3.9)	1.6 (0.5–4.9)	0.424		2.0 (0.4–9.1)	0.347		196	13 (6.6)	3.7 (1.5–9.4)	0.003		2.8 (0.9–9.5)	0.070	
55–64	171	10 (7.9)	3.3 (0.8–12.8)	0.075		3.8 (0.6–216)	0.145		144	4 (2.7)	1.5 (0.3–6.3)	0.589		1.1 (0.2–7.5)	0.916	
**Religion (n = 3071)**																
Protestant/Other Christian	1092	48 (4.5)	ref						1071	27 (2.5)	ref					
Muslim	53	3 (4.5)	1.0 (0.0–30.1)	0.997	0.791	1.2 (0.0–31.9)	0.923	0.714	121	7 (5.2)	2.2 (0.8–5.7)	0.103	0.144	1.2 (0.3–4.2)	0.773	0.689
No Religion	31	1 (2.7)	0.6 (0.1–2.8)	0.482		0.7 (0.2–2.8)	0.564		37	4 (9.5)	4.2 (0.9–18.9)	0.053		2.6 (0.5–12.2)	0.217	
Other	23	3 (9.3)	2.2 (0.4–11.4)	0.331		3.2 (0.5–15.8)	0.218		16	1 (5.6)	2.3 (0.7–7.4)	0.136		2.1 (0.6–7.9)	0.254	
Roman Catholic	322	22 (5.3)	1.2 (0.6–2.6)	0.63		1.1 (0.6–2.3)	0.707		305	8 (2.7)	1.1 (0.4–2.7)	0.844		1.1 (0.4–2.7)	0.849	
**Marital status (n = 2983)**																
Never married/ cohabited	199	5 (1.4)	ref						517	11 (2.3)	ref					
Divorced or separated or Widowed	479	23 (4.3)	3.2 (0.9–11.4)	0.056	0.065	2.4 (0.6–9.4)	0.174	0.172	175	5 (3.0)	1.3 (0.4–3.9)	0.609	0.187	0.6 (0.2–2.6)	0.497	0.679
Married/ cohabiting—monogamous	646	35 (5.4)	4.1 (1.3–13.7)	0.014		3.0 (0.9–9.8)	0.061		743	24 (3.0)	1.3 (0.6–2.9)	0.458		0.7 (0.3–2.0)	0.519	
Married/cohabiting—polygamous	145	11 (6.7)	5.2 (1.3–20.2)	0.012		3.7 (1.0–13.7)	0.037		79	5 (6.8)	3.1 (1.1–9.1)	0.03		1.1 (0.3–4.5)	0.848	
**Wealth quintile (n = 3071)**																
Highest	414	20 (4.4)	ref						505	7 (1.5)	ref					
Third	370	13 (3.9)	0.9 (0.4–2.0)	0.738		0.5 (0.2–1.4)	0.182		346	10 (3.0)	2.0 (0.8–5.3)	0.133		2.9 (1.0–8.0)	0.057	
Second	377	18 (4.8)	1.1 (0.5–2.5)	0.805		0.6 (0.2–1.8)	0.309		359	11 (3.2)	2.2 (0.8–6.1)	0.124		2.4 (0.8–6.7)	0.084	
Lowest	359	26 (5.9)	1.4 (0.6–3.2)	0.467	0.775	0.7 (0.3–2.2)	0.55	0.537	341	19 (5.2)	3.6 (1.4–9.3)	0.005	0.044	2.5 (1.0–6.1)	0.038	0.125
**Education level (n = 3071)**																
Complete Primary	379	20 (4.4)	ref						485	8 (1.7)	ref					
Incomplete Primary	950	43 (4.4)	1.0 (0.5–2.0)	0.874	0.414	1.0 (0.5–2.1)	0.961	0.393	640	22 (3.3)	1.9 (0.8–4.9)	0.122	< .001	1.3 (0.5–3.0)	0.588	0.018
No Formal Education	121	9 (7.7)	1.8 (0.6–5.2)	0.255		1.7 (0.7–4.4)	0.229		119	12 (10.9)	7.0 (2.7–18.4)	< .001		4.8 (1.5–15.7)	0.006	
**Ever had blood transfusion (n = 3068)**																
Yes	129	11 (9.0)	ref						65	1 (1.1)	ref					
No	1390	66 (4.3)	0.5 (0.2–1.0)	0.042	0.042	0.4 (0.2–0.9)	0.027	0.027	1484	46 (3.0)	2.8 (1.8–4.3)	< .001	< .001	3.2 (1.6–6.6)	0.001	0.001

Note: n = 3072 unless stated. For n<3072, the difference accounts for missing data due to non-response to the specific survey question

## Discussion

KENPHIA 2018 was the first nationally representative survey of HBV infection in Kenya that provided immediate test results to participants at the household. Our analysis reports an overall HBV prevalence of 3.0% (95% CI: 2.2–3.9%) among people aged 15–64 years. As expected, this HBV prevalence was somewhat higher than those estimated for Kenya in 2015 WHO estimate of 2.2% (95% CI: 1.6–2.9%) and the 2019 WHO estimate of 1.7% (95% CI: 1.4–1.9%) for the general Kenyan population, which included children <15 years old [2, 3]. These results suggest that HBV prevalence among children is lower than among adults and, when combined, it masks the higher prevalence in the adult population. In the same WHO estimates, Kenya was at a prevalence of 2.1% pre-HBV vaccination era, 0.9% in 2015, and 0.4% in 2019 among children <5 years of age [3].

We estimated the overall national burden of HBV infection as 800,100; (95% CI: 560,100–1,040,200), consistent with the 2019 WHO estimate of 892,500 (95% CI: 772,700–1,023,700) people [3]. Given that approximately 10–30% of people with HBV infection require treatment [4], we estimated that 80,000–240,000 people in Kenya, required HBV treatment in 2018. Overall, lack of formal education was significantly associated with HBV infection in our study. Additionally, being in the lowest wealth quintile was particularly associated with HBV infection among HIV uninfected persons, while rural residence and being married were independently associated with HBV infection among PLHIV. These findings highlight HBV infection in Kenya as a disease of the socioeconomically disadvantaged in society. This underscores the importance of implementing interventions against HBV infection under the umbrella of universal health care (UHC) and the ongoing free primary education programs rolled out by the Kenyan government. These will in part alleviate these factors associated with increased HBV morbidity in Kenya.

A meta-analysis of eight earlier studies (published before 23^rd^ October 2013) among different subpopulations estimated a 5.2% prevalence of HBV infection in Kenya [17], higher than the estimated prevalence in our analysis. Although the eight studies were not nationally representative, findings from our study suggest that HBV prevalence is likely decreasing, as suggested elsewhere in systematic reviews and meta-analysis [18, 19]. This highlightes the critical value of direct measures of national burden through nationally representative serosurveys to produce accurate estimates of HBV infection burden to inform programming towards elimination. The lower prevalence can also be attributed to a higher burden among older people with higher mortality, as well as declining rates [7] but also much lower infection rates among children and adolescents due to successful infant immunization. The overall HBV prevalence reported in this survey was somewhat lower than those documented among adults in several other national surveys in Africa: 5.6% in Zambia [20], 4.1% in Uganda [21], 3.9% in Rwanda [22], and 3.5% in Tanzania [23]. Overall prevalence observed in our study was about half the 2015 African region prevalence of 6.2% estimated by WHO [2], and lower than the West and Central African countries that had the highest estimated HBV prevalence in Africa [2], likely skewing the African region HBV infection estimates.

A trend analysis of WHO estimates suggests a significant reduction in the global prevalence of HBV infection (from 4.9% in the pre-vaccination era to 1.3% in 2015 and 0.9% in 2019) among children <5 years old [3]. These data suggest successful infant immunization programs that many countries including Kenya implemented in early 2000’s. However, estimates of the global HBV prevalence in the general population remained high; 5.0% in the pre-vaccination era, 3.5% in 2015, and 3.9% in 2019 [3]. Similar trends were observed in the Africa region; 8.3% in the pre-vaccination era, 3.0% in 2015, and 2.5% in 2019 among under five-year-old children. In contrast, among the general population; 9.0% in the pre-vaccination era, 6.1% in 2015, and 6.8% in 2019 [3]. Therefore, WHO HBV infection estimates for 2015 and 2019, compared to the pre-vaccine era [2–4], together with data from this study and other recent national surveys [20, 21, 23], further suggest that overall HBV prevalence is declining over time globally.

Our analysis documented regional variance in prevalence of HBV infections across Kenya. In 2018, the coverage of infant immunization in Kenya for the pentavalent vaccine (that includes HBV) was reported to average at 81% [24], but also varied widely geographically [24, 25]: among the poor, less educated, those residing in rural areas with limitted access to health facilities and those from urban informal settlements [24–27]. These could provide partial explanation to the observed varied geographical distribution of HBV infection in Kenya. Similar observations were made in the neighboring Uganda, where the estimated national HBV prevalence of 4.1% also varied geographically at subnational level, from 0.8% in the South-West to 4.6% in the Mid-North regions of Uganda [21].

This study reported lack of formal education as a factor significantly associated with higher risk of HBV infection. Higher prevalence of HBV infection among participants with low level of education was also reported by other PHIAs [21, 23]. Similar to our study, the high prevalence of HBV infection was observed among married/cohabiting participants in the Tanzania PHIA survey [23]. These findings suggest increased risk of HBV transmission to family members within most socioeconomically disadvantaged households, higher risk for horizontal transmission to sexual partners and potential risk for vertical transmission from mother-to-child within these families. This underscores the need for a combination of interventions to prevent both vertical and horizontal HBV transmission through increased community awareness, health education, early detection, treatment and vaccination. Robust prenatal screening programs and antiviral treatment of pregnant women with HBV infection is critical to reducing vertical HBV transmission. In this effort, WHO released Interim Guidance for Country Validation of Hepatitis Elimination in June 2021, which highlighted program target of ≥90% coverage for maternal antenatal HBsAg testing [28]. Additionally, preventative measures such as catch up vaccination for household contacts can reduce the likelihood of horizontal transmission. Education and access to these services among socially disadvantaged population as seen in this study is particularly important, as age of acquisition of HBV infection is inversely related to likelihood of developing chronic HBV infection.

We found higher HBV prevalence among PLHIV compared to their HIV-negative counterparts. Other national surveys in sub-Saharan Africa have documented similar findings among HIV infected persons: a prevalence of 4.7% in Uganda [21], 5.2% in Tanzania [23], and 7.0% in Zambia [20] in PLHIV, compared to a prevalence of 2.4% in Uganda, 3.4% in Tanzania, and 3.3.% in Zambia among HIV uninfected persons. These reports confirm the high HBV-HIV coinfection rates previously observed in meta-analyses: 9.9% in Africa [29], and globally at 8.5% [29], and 7.6% [30]. The prevalence of HBV infection found in this survey among HIV-negative participants was similar to 2.1% observed in 2007 among HIV-negative persons in Kenya [31]. The higher prevalence of HBV infection found among PLHIV with history of blood transfusion underscore the imprortance of proper screening of transfusable blood and blood products to guarantee safety against transfusible transmittable infections. However, higher prevalence of HBV infection observed in HIV-negative particpants without history of blood transfusion was rather unexpected and requires further study. The high HBV prevalence among PLHIV underscores the need for excellent synergy in implementing WHO HBV elimination goals [2] and UNAIDS’ HIV epidemic control targets for 2025 [32]. Exploring opportunities for integrated service provision sharing HIV program infrastructure to include HBV services would increase access to appropriate HBV care and treatment. For example, using laboratory diagnostic networks, clinical management clinics, human resources for health, commodity supply chain, health information systems, and community support groups set up for HIV, to provide HBV diagnostic and treatment services.

The 2017 Global Hepatitis Report placed Africa among the highest in viral hepatitis associated mortality in the world [2], yet knowledge of HBV status remains extremely low in most African countries. For instance, almost 90% of the 296 million people estimated to be infected with HBV in 2019 were unaware of their HBV infection status, and less than a quarter (22%) of the 30 million who knew their HBV infection status were on appropriate HBV treatment [4]. In a systematic review on pooled estimates for treatment eligibility per WHO and other guidelines, Tan and colleagues reported that one in every five HBV cases required treatment: 10% based on high HBV DNA level (>20 000 IU/mL), and 31% based on elevated abnormal alanine aminotransferase [33]. Although the median price of generic tenofovir disoproxil fumarate (TDF), a treatment for HBV, reduced by >85% from $208 per year in 2004 to $32 per year in 2016, treatment coverage remained less than 20% globally [6]. Therefore, the key to realizing WHO HBV elimination targets [2] in Africa, beyond availability of accurate diagnostics and affordable effective antiviral therapy, is an enabling policy environment at national levels ensuring increased equitable access to education, healthcare, diagnostic and treatment services.

Due to the high HBV prevalence among PLHIV in Kenya, patients receiving HIV treatment are put on TDF-based ART regimens—which have dual activity against both HIV and HBV [34]. The national ART guidelines further recommend maintaining TDF on any subsequent regimen revisions for HBV-coinfected PLHIV whenever HIV treatment regimen is switched due to virologic HIV treatment failure [34]. Therefore, through this programmatic intervention the 72.7% of HBV-coinfected PLHIV on ART found in this study were already on HBV treatment as part of their routine HIV therapy. However, the need for HBV treatment in Kenya remains unaddressed in the general population. The high absolute burden of HBV infection in the general HIV-uninfected population observed in this study, clearly suggests a need for increased access to TDF and inclusion of HBV treatment in UHC.

Although the implementation of WHO HBV treatment guidelines for limited-resource settings [8] seems plausible, the algorithm informing treatment decisions may prohibit access to HBV treatment in rural settings without robust networks of laboratory infrastructure and therefore require further simplification. Importantly, adapting simple HBV infection screening and treatment strategies would increase treatment coverage in sub-Saharan Africa, including Kenya, where we report high-risk geographic regions and risk groups [35]. When planning and rolling out HBV management programs among African countries lagging on implementation of the HBV elimination agenda, guidelines for scaling up HBV treatment should also emphasize prevention of cirrhosis by expanding treatment coverage to patients without advanced liver disease, as a key step towards reaching the 2030 WHO target on HBV-related mortality. Therefore, careful considerations integrating WHO recommendations and lessons learned from low-resourced countries [36–38], are needed in designing and implementing HBV elimination programs in Kenya and other resource-limited settings. Additionally, embracing the triple elimination agenda [9] to create synergy, would facilitate rapid scale up of HBV vertical transmission prevention efforts.

Findings from this study are critical in setting enabling policies for Kenya to achieve the WHO global hepatitis elimination goals by 2030 through accelerated implementation of targeted HBV diagnostic, treatment, and prevention services. These services include: routine screening of HBV infection in HIV programs to ensure those with HBV/HIV coinfection are initiated on appropriate treatment [39]; routine screening of pregnant women during antenatal care and timely initiation of antiviral therapy for those eligible; routine implementation of the birth-dose HBV vaccine in newborns, catch-up vaccination among young women and PLHIV, and completion of the vaccine series for all persons in a routine HBV vaccination program; and implemention of the triple elimination agenda (of HBV, HIV, and syphilis) to help overcome cost constraint of eradicating each infection separately [9, 40].

This study has several limitations. The study did not include virologic (nuclei acid amplification) confirmatory testing and therefore we could not generate estimates on HBV treatment gaps. HBV infection prevalence and burden estimates provided in this study were limited to adults aged 15–64 years in Kenya and cannot be extrapolated to the younger subpopulation aged 0–14 years old. Additionally, this survey did not collect data on HBV vaccination history. Although, Kenya introduced routine infant HBV immunization in 2004 as part of the expanded program on immunization, this program by design excluded catch-up vaccination for the older eligible population. Therefore, by excluding children (0–14 years) who are beneficiaries to the expanded infant HBV immunization, and by not collecting HBV vaccination data in this survey, we could not assess the impact of HBV immunization program in Kenya. While this study had a nationally representative sample size to estimate the national HBV prevalence, the study was underpowered to estimate HBV prevalence at the sub-national levels or within smaller strata. Furthermore, some regions had low HIV prevalence in the main survey, so the sampling of HIV-negative participants for the HBV sub-study likely yielded lower precision and wider confidence intervals for regional HBV estimates. Therefore, the regional estimates should be interpreted with caution, and the current 47 Counties (administrative regions) remain in need of such data for County level public health planning and programming. This study was not well-powered to estimate HBV prevalence among pregnant women. Such data is needed to inform policy formulation for interventions against HBV infection in this subpopulation to prevent vertical HBV transmission. The study weights were however explicitly designed to account for the study sampling design, allowing for the presentation of nationally representative results despite the over-sampling of HIV-positive respondents. Therefore, in spite of these limitations, the KENPHIA survey provided an important opportunity to report Kenya’s national population-based HBV prevalence estimates.

## Conclusions

We report data from the first nationally representative household survey in Kenya that estimated prevalence of HBV infection and HBV-HIV coinfection. A prevalence of 3% for HBV infection documented by this study in Kenya supports adoption and implementation of targeted national HBV screening, vaccination and care/treatment programs as recommended in WHO guidelines [4]. Implementation of the WHO strategy of triple elimination of HIV, HBV, and syphilis, while ensuring availability of the birth-dose of the infant HBV vaccine, screening for HBV infection among pregnant women and all HIV infected persons for treatment eligibility and provision of antiviral therapy for all infected persons, would significantly reduce HBV burden in Kenya and contribute towards the 2030 goal of HBV elimination. Kenya has an uneven geographic distribution of HBV infections. Therefore, targeting HBV treatment and prevention programs to areas with the highest-burden would achieve greater efficiency with limited resources.

## Supporting information

S1 Fig HIV testing algorithm KENPHIA 2018. (TIF)
