# Supplementary figures and images for: Prevalence of hepatitis B virus infection in Kenya: A study nested in the Kenya Population-based HIV Impact Assessment 2018
Source: PLoS One. 2024 Nov 14;19(11):e0310923. doi: 10.1371/journal.pone.0310923 (PMC11563396; doi:10.1371/journal.pone.0310923)

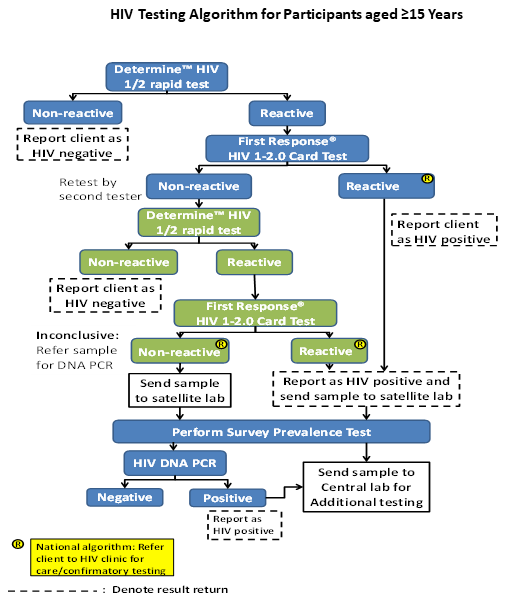

Supplement: S1 Fig — (TIF) [file pone.0310923.s001.tif]
